# Unleashing frugal innovation in private higher education institutions via intellectual capital: a systematic literature review

**DOI:** 10.12688/f1000research.73329.1

**Published:** 2021-11-03

**Authors:** Jayamalathi Jayabalan, Magiswary Dorasamy, Murali Raman, Murali Sambasivan, Sharbani Harun

**Affiliations:** 1 Faculty of Accountancy and Management, University Tunku Abdul Rahman, Kajang, Selangor, 43000, Malaysia; 2 Faculty of Management, Multimedia University, Persiaran Multimedia, Cyberjaya, 63100, Malaysia; 3 Asia Pacific University, Kuala Lumpur, 57000, Malaysia; 4 Thiagarajar School of Management, Madurai, 625005, India; 5 Technology Park Malaysia, Kuala Lumpur, 57000, Malaysia

**Keywords:** Intellectual capital, frugal innovation, IT capabilities, higher education institution, private university, systematic literature review, business sustainability, innovative paradigm

## Abstract

**Background:** Given the persistent challenges to the higher education business model, private higher education institutions (PHEIs) are exploring myriad ways to increase enrolment and income, while aggressively managing spending. Many PHEIs are facing financial distress and struggling because of decreasing budgets and declining revenue. Thus, carving unique strategies that direct the institution to focus on its core competencies, making additional budget cuts without compromising quality, developing new revenue streams, embracing new technology, and offering affordable programs, will ultimately lead to financial success. Frugal innovation (FI) can shed light on these challenges.

**Methods:** This paper presents a systematic literature review to investigate and analyse prior research that focused on FI within the sphere of intellectual capital (IC) and information technology capabilities (ITC) research, and their relationships in PHEIs. Transfield’s five phases were employed to extract journal articles published over a thirty-year period (1990 to 2020) from major online databases using keyword searches. Although an initial search generated 76,025 papers, the search for IC and FI yielded 41 papers, and finally only two papers were selected as they clearly related IC with FI.

**Results**: There was a research gap in the literature published from 1990 to 2020 regarding IC applications to achieve FI. This work revealed that IC and ITC research for FI in PHEI remain insufficiently explored.

**Conclusions: **Further research is required on the evaluation model of IC, ITC and FI, methodologies, empirical analysis, and the development of measurement metrics. A limitation to this study is the number of keywords selected.

## Introduction

Over the recent decades, higher education institutions (HEIs) have been challenged by the shifting landscape of employment, technologies and demand. In the globalisation era, HEIs require a paradigm shift which is expected to focus more on ‘global, digital and information’-based rather than being conventionally ‘national, analogue, industrial economy’-orientated. ^1^ HEIs play a critical role in the knowledge-based society and serve as a reservoir of knowledge. Recently, HEIs faced considerable competition and challenging situations to gain a competitive advantage in national and international settings. In particular, private higher education institutions (PHEIs) encounter considerable challenges to strike a balance between maximising shareholders’ financial growth and improving educational quality. Many local PHEIs are encountering difficulties to persist in the industry, with 53% of PHEIs incurring losses before taxes and 55% incurring losses after taxes. ^2^ This tremendous fiscal strain has caused various impacts including of job losses and learning disruptions for approximately 5,800 academic staff and 121,000 students in Malaysia, given the inadequate quality of education in unprofitable institutions. ^2^

In addition, PHEIs have been blooming in Malaysia to meet the increasing population demand. ^3^ As a result, intense competition is apparent among PHEIs. PHEIs are transforming education into a business model where the curriculum and programmes are tailored to accommodate the masses and are expected to generate high commercial value. Reductions in funding will have implications on the quality of education. ^4^ An urgent need exists for quality revolution in PHEIs, and for building a 21st century model to adapt to current social and technological changes. PHEIs should be ready to explore and innovate their curriculum design to produce graduates with high competency and skills. Furthermore, PHEIs must secure additional resources to finance their modernisation and development in a period of tight or decreasing public budgets, without forcing students to pay for the benefits of higher education which accrue to society at large. ^5^^-^^7^

Recently, PHEIs have been experiencing unexpected challenges because of limited budgets, declining revenues, resources constraints and increasing cost. ^8^ Therefore, business sustainability has become the main issue for the higher education system. Sazonov *et al.* ^9^ asserted that only HEIs with stable and sound financial positions will be able to persist and meet the requirements of their various stakeholders, to provide high quality yet equitable and affordable education while maximising shareholder wealth. Additionally, PHEIs face pressure from industry and academia to boost innovation through alternative approaches in a resource-constrained environment without considerable investment, ^10^ and provide solutions that are substantially more affordable. ^11^ Therefore, financially challenged PHEIs are adopting low-cost approaches which require new innovative paradigms such as frugal innovation (FI). ^12^

Most importantly, a paradigm shift in the mindset and approach among leaders is needed and will require PHEIs to respond to a rapidly changing environment, by aligning their strategies to meet the current landscape. ^13^^,^^14^ Moreover, according to, ^15^^,^^16^ private education has been the main contributor to the nation’s economic development and gross domestic product (GDP). A consistent increase in GDP contribution has arisen between 2015 and 2019, at RM14.09bil, RM14.84bil, RM15.70bil, RM16.62bil and RM30bil. ^17^ Moreover, PHEIs must focus on core functions, create closer integration with industry, collaborate with local and international communities and promote greater efficiency in operations.

Intellectual capital (IC) is an emerging issue for academics, governments and investors ^18^ and has been studied in developed countries, as it is regarded as a crucial indication of organisational development in a knowledge economy. ^19^ Youndt *et al.* ^20^ asserted that, in knowledge-intensive organisations such as universities, prudent management of IC can ensure that procedures are successful and that entities can produce value. IC is vital in improving PHEI performance, innovation and creativity. ^21^^,^^22^ Several calls have been made for research on improving the management of IC in PHEIs. ^23^^,^^24^ However, studies that focuses on IC management and resource efficiency in PHEI are still limited. ^25^^,^^26^

FI involves managing the entire value chain with limited physical and financial resources, resulting in improved product quality and cost savings. Therefore, a company can benefit through cost reduction, because of prudent resource management, full utilisation of existing components, adoption of cost-effective technology, and simplified design. ^27^^,^^28^ By adopting FI, PHEIs will be able to meet stakeholder expectations and eventually lead to increased profitability and sustainability. Therefore, developing a resource-saving agenda and services, by aiming at core elements and avoiding wasteful spending, is crucial in a resource-constrained environment. FI is observed as ‘core functionality’, is performance-based, focuses on usage of resources, ‘ruggedisation’, cost saving, ‘no-frills strategy’ and environmental concern. ^12^^,^^29^^-^^32^

Information technology capability (ITC) supports innovation processes for increasing productivity, improving customer relationships and lowering operating expenses. An organisation’s success is not solely contributed to by investing in an IT system, but also arises from the ability of firms to acquire ITC in an ever-changing business environment. Brown and Sambamurth ^33^ defined capability as ‘the distinctive organisational skills for combining available resources and sustaining superior performance’. Thus, ITC refers to particular resources, skills, information, knowledge, procedures and relationships that empower companies to viably acquire, adopt and control IT applications and administrations, to gain innovations and superior performance. ^34^ As such, a PHEI with efficient IC management can develop strong ITC, as the institutions would be able to scan the environment and sense, monitor and strengthen their organisational capabilities.

IC, ITC and FI together will be able to provide new opportunities for PHEIs to redesign their business processes and model. Therefore, this gap prompted the researchers to investigate and analyse prior research that focused on FI, within the sphere of IC and ITC literature and their relationships in PHEIs.

Given this backdrop, the research questions for this study were as follows:

1. How did the corresponding literature of IC and FI in PHEIs evolve?
2. What are the research gaps on the influence of ITC on IC and FI in PHEI?
3. What are the possible future research directions?

The objectives of this study were as follows:

1. To identify the main areas of IC and FI in the PHEI context.
2. To analyse research gaps in the literature on the influence of ITC on IC and FI.
3. To identify the possible future research directions.

Hence, the main contribution of this paper is to fill the gap in existing literature on PHEI, underlining the growing importance of IC and FI. In addition, the researchers aim to provide a review on past studies related to applying IC to achieve FI in PHEI. This paper is organised as follow: first, the scope of this research is explained; the following section describes the method to collect and compare the existing literature; the Results section represents keyword search result, and gap analysis; the Discussion provides avenue for future research, and the Conclusions section outlines the conclusions of this research.

## Methods

**Figure 1.**
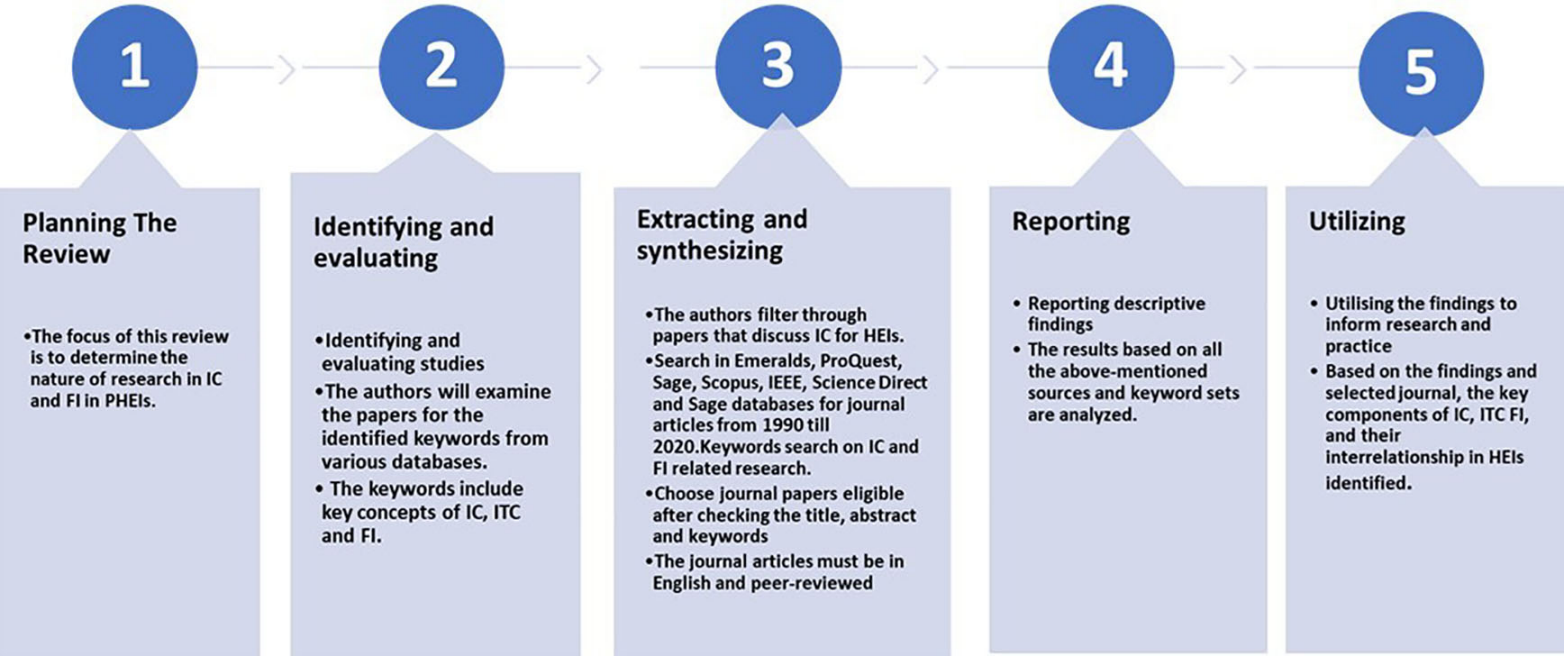
Five stages of systematic literature review.

**Figure 2.**
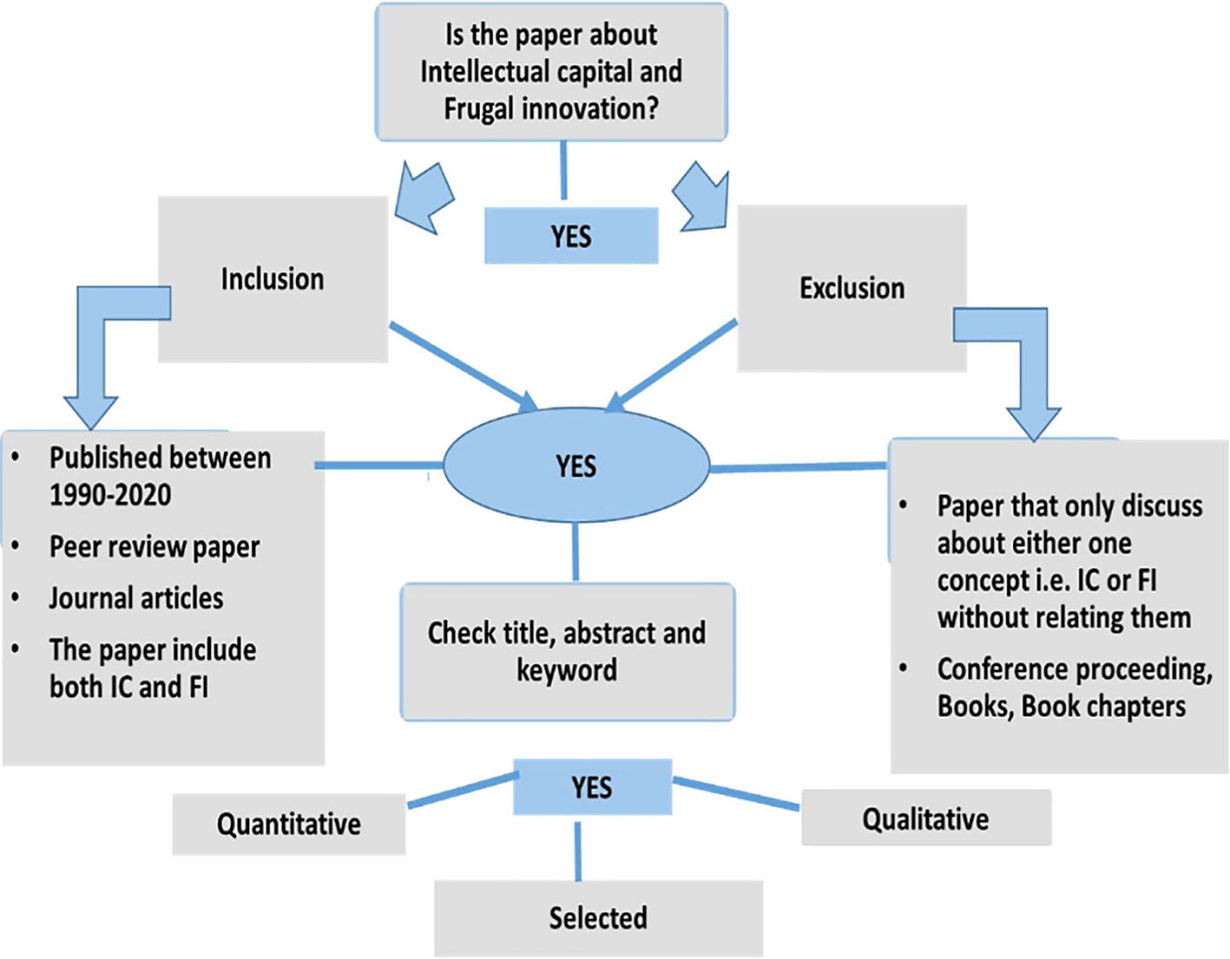
Inclusion and exclusion criteria.

**Figure 3.**
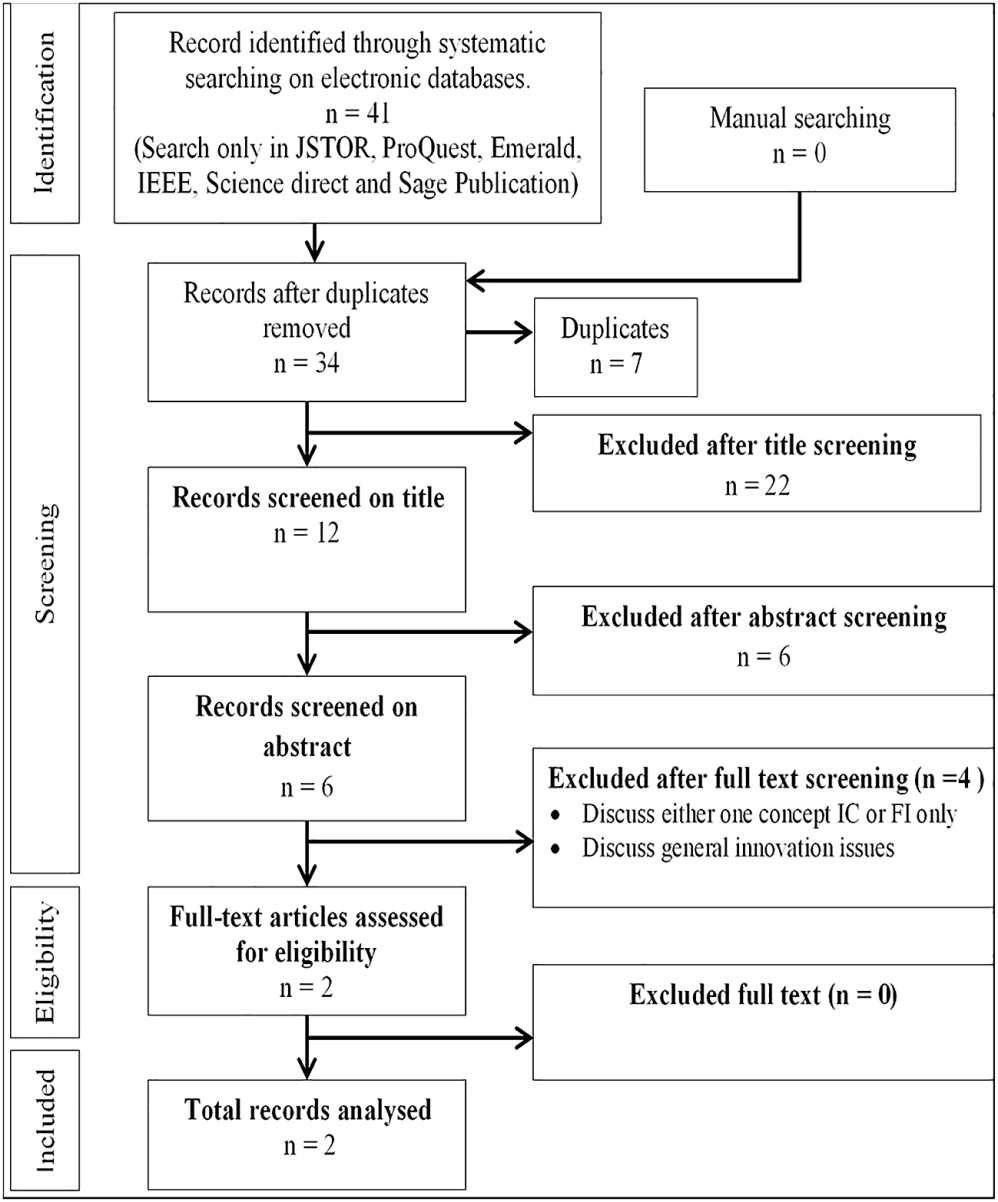
PRISMA extraction flow.

This paper was designed to present a systematic literature review on effects of IC on FI. Our assessment of the literature was based on the five steps of systemic review that were presented by, ^35^ which entails five phases as shown and described in Figure 1. Major online databases that were selected include IEEE, Sage Publications, Emerald, JSTOR, Scopus, ProQuest, and Science Direct. The search technique employed in each database differed depending on the terms and the capacity to combine the terms using Boolean operators, and the usage of truncations such as ‘intellectual capital’ AND ‘information technology capability’ AND ‘frugal innovation’ AND ‘Higher education institution’ OR ‘higher learning institution’ OR ‘university’ OR ‘institute of higher learning’. The final search method was customised for each database to optimise the retrieval of relevant research. Each article was reviewed for eligibility after checking title, abstract and keyword. Two authors (JJ, MD) independently filtered papers that discussed IC and FI. Any disputes about study inclusion were solved by negotiations among the authors until consensus was reached. The papers analysed were further selected based on inclusion and exclusion criteria as shown in Figure 2, and the selection process is shown in Figure 3 (Prisma extraction flow). Studies were included if they were (i) published between 1990 and 2020, (ii) peer-reviewed journal articles and (iii) focused specifically on IC and FI. Studies were excluded if they were (i) discussing only one term i.e., either IC or FI without relating them, (ii) not published in English and (iii) books, conference proceedings, dissertation or magazines. Prior to the screening procedure, duplicate studies were eliminated. Relevant data were extracted and summarised in tables based on study design, paper type, methodology and publication trend by year. There are a few limitations to this systematic study that should be mentioned. First, because the articles included were only acquired from journal publications, the data on the IC and FI may be incomplete. Second, the review only considered English-language publications. Several publications discovered in this review were in foreign languages, however they were removed as the authors did not have the capacity to comprehend them.

During the data charting, we will test a predefined data extraction sheet that has been accepted by consensus of authors (JJ, MD). We will extract year of publication, source of information, design and study-specific information, and findings that address our research questions. The first author (JJ) extracted the data while the second author (MD) carried out a cross-check to provide an overview of the extracted data items in the results. Results are presented graphically. JJ provided a summary of the study's features based on both abstract and full-text screening and a random check conducted by the second reviewer. The full spreadsheet listing all extracted items was stored in a database and included in the ‘Data availability’ section and reference list. Figure 3 illustrates the process of document selection based on title and abstract, followed by full text selection. Only published, peer-reviewed journal articles were considered while other publications such as newspaper articles, books and conference proceedings were not included.

### Ethical considerations

This study was approved by the Research Ethical Committee (REC) of the Multimedia University (EA1362021). The study was conducted according to the guidelines of and was approved by the REC.

## Results

The authors used a thematic analysis, summarizing the results by year, source database, methodology used in the study and classification by type of paper. A total of 76,025 papers was obtained with the keyword ‘intellectual capital’. For the keywords search of ‘information technology capability’ and ‘frugal innovation’, 14,122 and 2,320 journal papers were listed, respectively. However, when the researcher entered a combination of ‘intellectual capital’, ‘information technology capability’ and ‘frugal innovation’ keywords, no journal was found. Similarly, when the researcher used the keyword sets ‘intellectual capital’ + ‘information technology capability’ + ‘frugal innovation’ + ‘Higher education institution’ or ‘higher learning institution’ or university or ‘institute of higher learning’, no papers were obtained.

### Publication per year

The authors selected journal articles from five main databases from the year 1990 to 2020 ( Figure 7). The authors analyzed the trend of publication over the years for IC and FI (34 articles). Increasing trends were observed in terms of number of publications over the years ( Figure 7). There was also a drastic increase during the year 2020, which indicates the topic gained popularity.

### Publication by methodology

From the collection of articles, this section aims to discuss the main methodology applied by each paper. Table 3 describes the themes associated to each publication based on whether they used quantitative methods (survey or archival), qualitative (case study approach or observation), or conceptual model approaches (literature review studies or theoretical concept). As summarized in Figures 5 and 6, approximately 44% were empirical papers, followed by conceptual papers (41%) and reviews (15%).

###  Publication by source database

**Figure 4.**
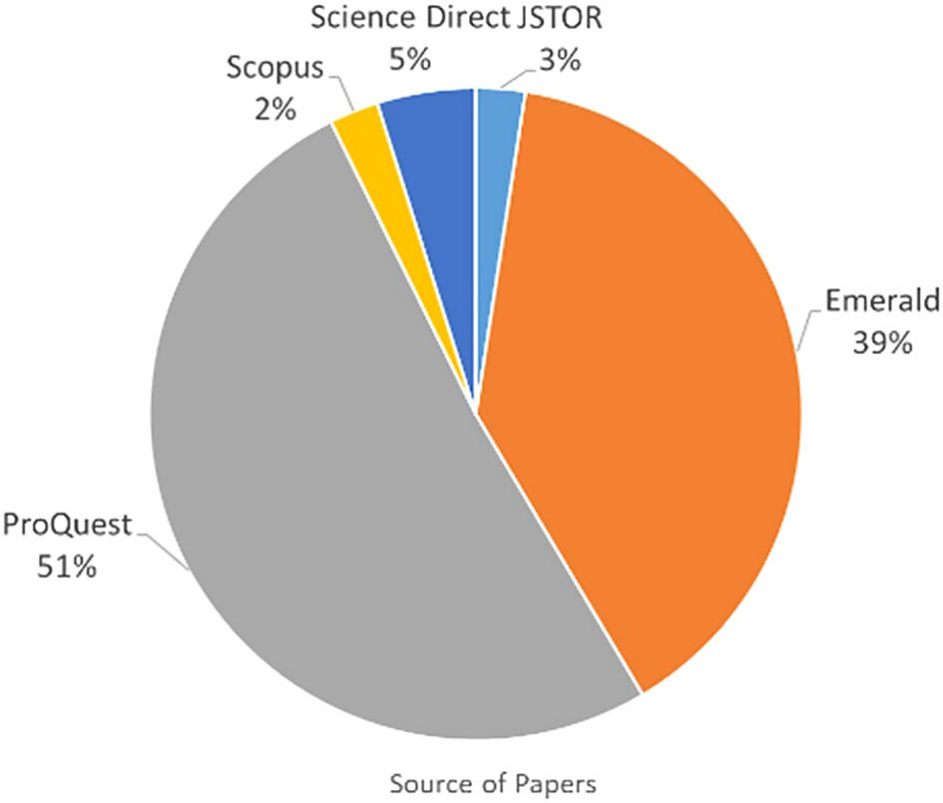
Percentage of 41 papers by source database.

The articles were grouped according to whether they were obtained from searching IEEE, Sage Publications, Emerald, JSTOR, Scopus, ProQuest, and Science Direct to analyse the one yielding the most publications. Paper grouping per database is presented in Table 2 and Figure 4. As shown in Table 1, 41 papers were listed for IC and FI. The 41 papers were obtained from five online databases as shown in Table 2 and Figure 3. Most papers were published in Emerald and ProQuest. Only 34 papers were related to IC and FI from the total of 41 papers. The seven remaining papers were duplicates which appeared in multiple online databases and were removed from the analysis. Further investigation of the 34 papers revealed only two papers related to IC and FI. ^36^^,^^37^ The search using online databases did not exclusively list papers with the given keywords. Therefore, the selection criteria involved accepting all papers that discussed IC and FI ( Table 1) and rejecting those that discussed either IC or FI without relating them to each other. The search for IC and FI yielded 41 papers but only two clearly related IC with FI. Referring to Table 2 and Figure 4, 51% of the papers were from ProQuest database, followed by Emerald (39%).

**Figure 5.**
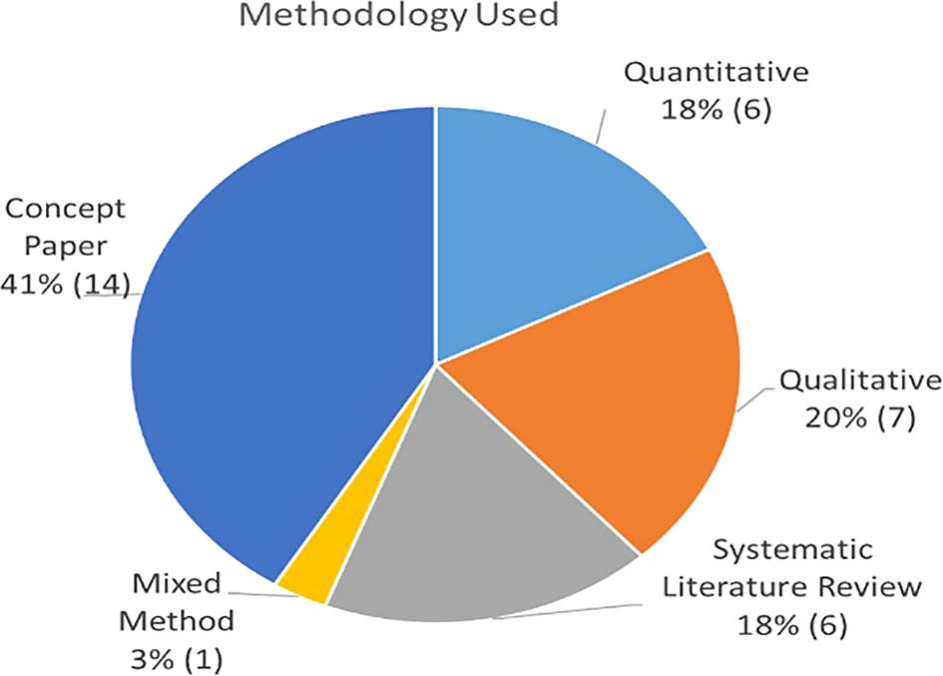
Percentage of 41 papers by source.

**Figure 6.**
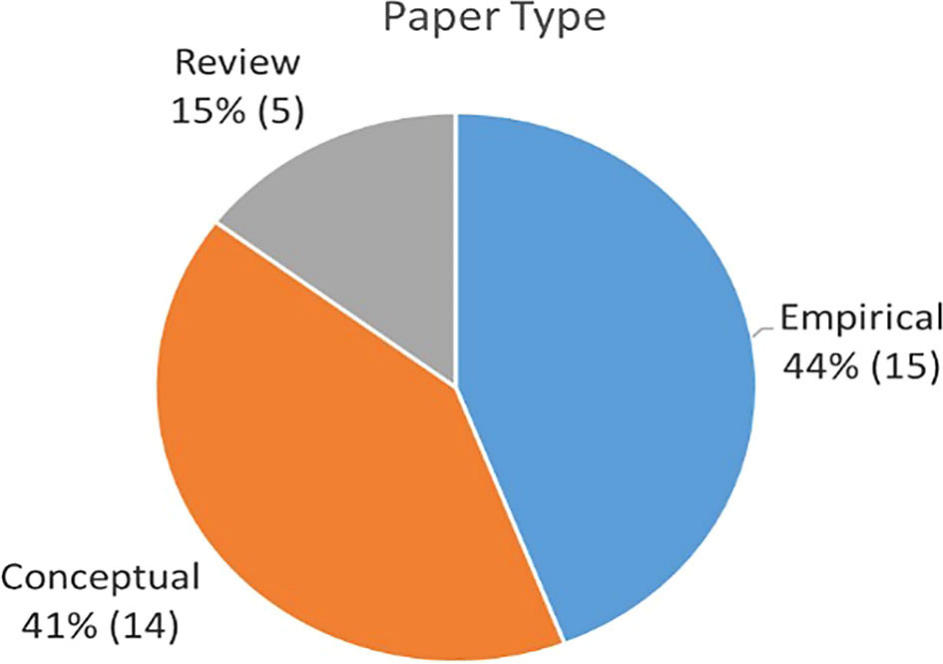
Percentage of paper by type.

**Figure 7.**
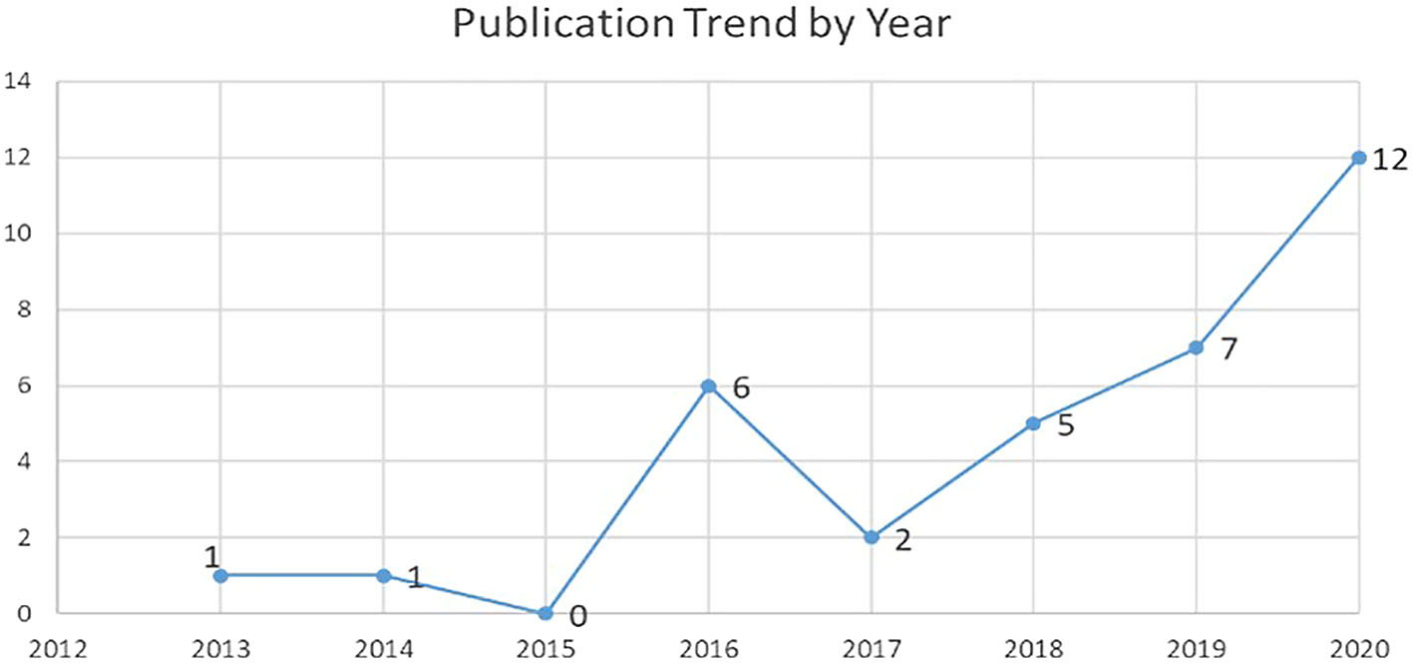
Publication trend by year.

**Table 1.** Summary of keyword search results.

No.	Online database	Intellectual Capital	Keywords combinations								
			Intellectual Capital AND Higher education Institution or Higher learning Institutions or universities or Institution of higher learning	Information technology Capability	Intellectual Capital AND Information technology Capability AND Higher education Institution or Higher learning Institutions or universities or Institution of higher learning	Frugal innovation	Frugal innovation AND Intellectual Capital	Intellectual Capital AND Information technology Capability AND Frugal innovation	Intellectual Capital AND Information technology Capability AND Frugal open innovation	Intellectual Capital AND Information technology Capability AND Frugal innovation AND Higher education Institution or Higher learning Institutions or universities or Institution of higher learning	Intellectual Capital AND Information technology Capability AND Frugal Open innovation AND Higher education Institution or Higher learning Institutions or universities or Institution of higher learning
1	JSTOR	181	146	12522	0	67	1	0	0	0	0
2	Emerald	8246	229615	619	121719	552	16	0	0	0	0
3	ProQuest	19768	7,027,625	763	0	394	21	0	0	0	0
4	Scopus	3946	172	70	0	237	1	0	0	0	0
5	IEEE	17	0	0	67	6	0	0	0	0	0
6	Science Direct	4989	1	127	0	1029	2	0	0	0	0
7	Sage Publications	38878	849499	21	0	35	0	0	0	0	0
	Total:	**76025**	**8,107,058**	**14122**	**121786**	**2320**	**41**	0	0	0	0

**Table 2.** Source of 41 Papers.

Online database	No of papers	Percentage	Selected for this study
JSTOR	1	2%	0
Emerald	16	39%	1
ProQuest	21	51%	1
Scopus	1	2%	0
Science Direct	2	5%	0
Total	41	100%	2

From a comparison of the articles published over the years, research on IC and FI was found to be discussed in 34 papers after removing duplicate publications. Further screening on titles resulted in the selection of 12 papers relevant to the research topic. Furthermore, the authors’ abstract screening led to only six papers being selected while others were excluded, as they were not widely discussing the research topic. Finally, full-text screening was carried out by the authors, and it appeared only two papers appeared had carried out relevant research on the topic. The remaining four articles excluded were either discussing only one of the concepts (IC or FI) and no relationship was found to be explored between the two, or they discussed general issues in innovation (as shown in Figure 3). Of the two papers ( Tables 3 and 4), Dost *et al.* ^36^ investigated the effect of internal and external sources of knowledge management on FI, which is moderated by technological turbulence and market turbulence. Bencsik *et al.* ^37^ wrote a conceptual paper on FI and knowledge management. The goal of FI does not only focus on providing low-cost products and services, but rather on establishing a flexible thinking to uncover human knowledge, and the capability and usage of internal or external technologies, resulting in lower innovation cost of processes and products. ^38^ Thus, this study aims to enhance the understanding of the components of IC: ‘human capital’, ‘structural capital’, and ‘relational capital’ as main factors in the generation of FI through ITC.

**Table 3.** Descriptive information of the 34 core papers.

No	Author	Year	Source database	Respondents	Methodology	Type of paper
1	^52^ Abbas et al. (2019)	2019	Proquest	SMEs in Pakistan	Quantitative	Empirical
2	^53^ Arshi et al. (2020)	2020	Emerald	713 entrepreneurs in India, Oman and the United Arab Emirates	Quantitative	Empirical
3	^54^ Bashir and Farooq (2018)	2018	Emerald	Nil	Systematic Literature Review	Empirical
4	^37^ Bencsik et al. (2016)	2016	Proquest	Hungarian Firms	Nil	Conceptual
5	^55^ Bhattacharya et al (2020)	2020	Emerald	Construction companies in India	Qualitative	Empirical
6	^56^ Botha (2019)	2019	Emerald		Qualitative	Conceptual
7	^57^ Brem et al. (2014)	2014	Proquest	Nil	Nil	Conceptual
8	^58^ Deborah (2016)	2016	Science direct		Nil	Conceptual
9	^59^ Dhamija & Bag (2020)	2020	Emerald	Nil	Systematic Literature Review	Review
10	^60^ Diaw et al. (2020)	2020	Proquest	African Industry	Nil	Conceptual
11	^36^ Dost et al. (2019)	2019	Emerald	SMEs	Quantitative	Empirical
12	^61^ Durst et al. (2018)	2018	Emerald	671 Turkish firms operating in five industries	Quantitative	Empirical
13	^62^ Farooq (2020)	2020	Emerald	Organisation	Systematic Literature Review	Review
14	^63^ Gupta et al. (2016)	2016	Scopus	Nil	Nil	Conceptual
15	^64^ Gupta (2019)	2019	Proquest	Nil	Nil	Conceptual
16	^65^ Hart et al. (2016)	2016	JSTOR	nil	Nil	Conceptual
17	^66^ Herting et al. (2020)	2020	Proquest	Nil	Qualitative	Empirical
18	^67^ Horn & Brem (2013)	2013	Proquest & Emerald	Nil	Systematic Literature Review	Empirical
19	^68^ Hossain (2017)	2017	Proquest & Emerald	Nil	Systematic Literature Review	Review
20	^69^ Hossain et al. (2016)	2016	Emerald	Nil	Systematic Literature Review	Review
21	^70^ Kabadurmus (2020)	2020	Emerald	Business Enterprise	Qualitative	Empirical
22	^71^ Koerich et al. (2019)	2019	Proquest	Nil	Nil	Conceptual
23	^72^ Kok (2017)	2017	Proquest	3 China Banks	Qualitative	Empirical
24	^73^ Liotta et al. (2018)	2018	Proquest	Health	Nil	Conceptual
25	^74^ Michelam et al. (2020)	2020	Proquest	Nil	Nil	Conceptual
26	^75^ Nag et al. (2020)	2020	Proquest	Nil	Nil	Conceptual
27	^76^ Penco et al. (2020)	2020	Emerald	Italian SME - F&B Industry	Quantitative	Empirical
28	^77^ Rese et al. (2020)	2020	Proquest & Emerald	95 German workers	Quantitative	Empirical
29	^78^ Sahay (2016)	2016	Proquest	Public health	Nil	Review
30	^79^ Snehvrat & Dutta (2018)	2018	Emerald	NA	Qualitative	Empirical
31	^80^ Hosung et al. (2018)	2018	Proquest	Korean manufacturing	Mixed Method	Empirical
32	^81^ Sordi et al. (2019)	2019	Emerald	46 entrepreneurs	Qualitative	Empirical
33	^82^ Yin et al. (2019)	2019	Science direct	China Firms	Nil	Conceptual
34	^83^ Zhifeng et al. (2020)	2020	Proquest	Chinese Enterprise	Nil	Conceptual

**Table 4.** Analysis of 34 core papers by research objectives, variables, moderator, mediator and findings.

No	Author	Research objectives	Intellectual capital variables	Mediator	Moderator	Findings
1	^52^ Abbas et al. (2019)	To investigate the link between corporate social responsibility and firm performance through the moderating role of the social media marketing application in small firms.	Corporate social responsibility (CSR), social media marketing	Social media technology application		Customers' engagement and firm performance can be enhanced for firm that focuses on CSR practices when social media intergrated with marketing strategies.
2	^53^ Arshi et al. (2020)	To propose 'Start-up Evaluation Calculus Using Research Evidence' (SECURE) business model to allow the measurement of the impact of business model design on start-up performance in emerging economies.	Business model			It is suggested that the business model framework is required for businesses with ex ante indicators shows positive impact to firm performance.
3	^54^ Bashir and Farooq (2018)	To provide a systematic review of the linkage between 'knowledge management', 'business model innovation' and 'firm competence'.	Knowledge management'			The integration of knowledge management and business model innovation leads to a sustainable competitive advantage.
4	^37^ Bencsik et al. (2016)	to show the relationship between frugal innovation and knowledge management.	Knowledge management, knowledge sharing			Hungarian companies have shown that there is relationship between 'human,relationships,knowledge and creativity' and the way of thinking.
5	^55^ Bhattacharya et al (2020)	To give suggestion of a conceptual framework on the enablers of growth and performance metrics.	human and operational capabilities			Capabilities for 'operational and process excellence,' 'unique products and services,' and 'visionary leadership' highest ranked enabler of growth.
6	^56^ Botha (2019)	To suggest future evolution of 'innovation from a human only initiative, to human–machine co-innovation, to autonomous machine innovation'.	innovation from a human only initiative			Evolution of Innovation is from a 'human-only activity, to human–machine co-innovation, to incidences of autonomous machine innovation, based on the growth of machine intelligence and the adoption of human–machine partnership management models in future'.
7	^57^ Brem et al. (2014)	To provide overview of the following terms 'jugaad, frugal innovation, frugal engineering, constraint-based innovation, Gandhian innovation, catalytic innovation, grassroots innovation, indigenous innovation, and reverse innovation.'				Research and development (R&D) in developed market firms (DMFs)' framework in emerging markets.
8	^58^ Deborah (2016)	To explore how organizational fields developed from local entreprenuers' effort to initiate their start-ups.	Organisational entrepreneur			Organizational field formation influenced by an entrepreneur to become supportive under some circumstances, move innovation through the legitimation stages of local validation, diffusion and general validation'
9	^59^ Dhamija & Bag (2020)	To review past literatures artificial intelligence.	Artificial intelligence			6 clusters: 'Artificial Intelligence and Optimization, Industrial Engineering/Research and Automation, Operational Performance and Machine Learning; Cluster, Sustainable Supply Chains and Sustainable Development, Technology Adoption and Green Supply Chain Management and Internet of Things and Reverse Logistics' were identified.
10	^60^ Diaw et al. (2020)	To explore the industrialization strategy advantages at 'manufacturing entry-level products'.	Potential of industrial development			Entry-level product lines play a crucial role in industrialization of Africa
11	^36^ Dost et al (2019)	To examine the relationship between internal and external sources of knowledge on frugal innovation (FI), through the moderating 'role of market and technological turbulence'.	Internal and external source of knowledge'		Technological turbulence and Market turbulence	There is a relationship between internal and external sources of knowledge on FI. 'The moderation of technological turbulence strengthens the effects internal and external sources of knowledge had on FI. Market turbulence strengthened the effects of external sources of knowledge but surprisingly weakens the effects of internal sources of knowledge on FI'.
12	^61^ Durst et al. (2018)	To investigate how the different factors effect the new product development process in Turkish firms.	Human capital and organisational capabilities			Human capital, leadership, marketing capabilities, and business and institutional networks shown some differences in terms of the commercialization of newly developed products in domestic and international markets.'
13	^62^ Farooq (2020)	To investigate the relationship between knowledge management and value creation.	knowledge management (KM)		Social capital	Knowledge management able to influence value creation by having good interpersonal relationships with all stakeholders by managing 'social capital through knowledge management processes'.
14	^63^ Gupta et al. (2016)	To plead for more 'reciprocal, respectful and responsible exchanges of knowledge between formal and informal sector adding value to the contributions of grassroots green innovators'.	Relational or social capital			Conventional understanding of open innovation theory is highly inadequate for dealing with emerging challenges in leveraging contingent conditions of climate risks, asymmetry of knowledge and power and lack of reciprocity and responsibility among the formal and informal actors.'
15	^64^ Gupta,(2019)	To support 'research propositions on the various proposed relationships in the GRI framework'.	Grassroots innovation			To develop framework to show the relationship among the contributing factors of "feasibility and value of GRI in society".
16	^65^ Hart et al. (2016)	To propose a set of construct to predict successful BoP business innovation.	BoP business innovation			BoP for poverty alleviation and sustainable development.
17	^66^ Herting et al. (2020)	To find out how 'discruptive dynamics' able to effect business models with different items from practitioner's view.	Disruptive dynamics			developed the 'business model components,' From practioner viewpoint.
18	^67^ Horn & Brem (2013)	To identify from the past research the new emerging themes in innovation management.	Innovation management			Seven major fields for future research in innovation management theory and practice: customer orientation, network organisation, sustainability, frugality, intellectual property, business model and global innovation'.
19	^68^ Hossain (2017)	To provide literature review on business model innovation (BMI) and suggest upcoming research work that can be done.	Business model innovation			the findings identified most important themes and logical arguments in the current research.
20	^69^ Hossain et al. (2016)	To provide extensive literature review on the evolution of open innovation.	open innovation			Proposed a framework on open innovation through analysis of past gap.
21	^70^ Kabadurmus (2020)	To investigate the relationship between 'organizational and environmental (competition, capital scarcity and organization of labor) factors and firms’ innovation activities within the supply chain'.	organizational resources capability			There is a relationship between strategic resources and firm's competitive advantage which also depends on type of innovation activities in SCM.
22	^71^ Koerich et al. (2019)	to study on evolution of frugal innovation and the trends in current literaturesand suggestion on further work to be done.	Evolution of Frugal innovation			development of few 'measurement tools for frugal innovation'.
23	^72^ Kok (2017)	to examine the approach of 'Financial innovation' in large commercial bank in China.	Bank's innovation strategy and local government policy			The study found that regulation can hinder financial innovation and it is depending on whether bank's innovation strategy coherent with local government policy.
24	^73^ Liotta et al. (2018)	To propose a 'model for building African health capacity through regional health innovation networks through commercialization of health technologies'.	Network based Health innovation strategy			the study found that building business operation capacity and local legal aspects are very crucial for 'product-focused, network-based health innovation strategy'.
25	^74^ Michelam et al. (2020)	To examine extensive literature review on the main aspects of KBUD in promoting smart sustainable cities.	KnowledgeBased Urban Development (KBUD) and smart and sustainable cities			proposed 'multi-dimensional and integrated approach for smart and sustainable urban development.'
26	^75^ Nag et al. (2020)	To evaluate techniques and methods to achieve 'Blue Ocean Strategy'.	Blue ocean Strategy			The study shown that management operational efficiency will help to firm to achieve competitive advantage by applying the 'principles of Operations Management'.
27	^76^ Penco et al. (2020)	To investigate the effect of 'strategic orientation (defined using the Miles and Snow’s paradigm) on the processes of strategic decision-making and organisational design in medium-sized firms (MEs) in Italian family food and beverage industry (F&B)'.	impact of strategic orientation on the processes of strategic decision-making and organisational design in medium-sized firms			To describe the use of the 'prospector orientation in modifying the behavioural models among the selected companies."
28	^77^ Rese et al. (2020)	To examine factors influence 'knowledge sharing which focuses on attitude, behavior and individual creativity.'	Knowledge sharing and sharing behaviour			there is a relationship between 'knowledge sharing attitude and actual sharing behavior in CWS improve coworkers’ creativity.'
29	^78^ Sahay (2016)	To investigate the drivers and challenges of big data in health system.	Big Data and health systems			highlighted the key 'implications for LMICs governments on the potential of big data to address public health concerns'.
30	^79^ Snehvrat & Dutta (2018)	To study on 'multi-faceted role of metaroutines in dealing with nested ambidexterity challenges experienced during new product introductions (NPIs) at Tata Motors, an Indian automotive giant'.	ambidexterity dynamics across strategic', 'business unit' and 'functional levels'.			"The role of embedded NPI metaroutine aspects in promoting multi-level ambidexterity offers a distinct form when compared with other academically established forms of structural, contextual and temporal ambidexterity".
31	^80^ Hosung et al. (2018)	To investigate the 'role of social entreprenuership,product innovation attributes and social capital on value creation and financial performance'.	Social entreprenuership	Social capital		Social entrepreneurship has relationship with product innovation and social capital in SEs and products’ simplicity, usability and standardization positively affect the social value creation of Ses'.
32	^81^ Sordi et al. (2019)	To examine the 'exaptation creation capability with practitioners and academics in the field of management' and ability to generate organisational performance.	exaptation - capability to generate performance for firms			the study found '13 cases of exaptation associated with 9 different kinds of organizational entities'.
33	^82^ Yin et al. (2019)	to compare 'rural and urban innovation system and propose new theoretical structural model'.	rural and urban innovative system			The study proposed all the 'future challenges in fostering a strong rural innovation system'.
34	^83^ Zhifeng et al. (2020)	To explore the development of 'Chinese style innovation' by analysing past studies and propose future work.	Development of Chinese-style innovation			the study found relationship amongst the 'New Economic Era, Chinese-style innovation and the International Repercussions'.

Some of the challenges faced throughout the search included selecting the right keywords and terms during the data extraction process, where there is a risk of either too much or not enough results being obtained, depending on the keywords use. Apart from that, the description of inclusion and exclusion criteria can have practical implications too. Finally, ‘low recall’ for keywords such as IC, ITC and FI altogether seems to be not appearing in HEIs context. Finding the optimum combination of keywords and adjusting the precision to maximize system accuracy is difficult. The goal of this systematic reviews was to include knowledge from all relevant research. Some of the limitations include a lack of information from some research, which can jeopardize the validity of a review. This is due to inadequate data due to only a few studies being published, or because of insufficient reporting within a published article. These issues fall under “publication biases”, even though these biases are due to unpublished comprehensive research and the publication of selected results in relation to authors’ conclusions.

This study adds to the improvement of IC research in the education sector, in which experiences from FI play an essential role in the growth of HEIs’ performance. The present article classification can assist future scholars in reviewing references based on their study requirements. Future research can utilize this literature review as a starting point to further comprehend FI to obtain quality literature. In the field of IC, scholars may involve more information system application, as well as Artificial Intelligence in their future work including system development, methodologies and strategies for PHEIs.

## Discussion

### Evolution of IC and FI research

Prior research has thus far considered the key components of IC and its measurement indicators. PHEI performance levels are measured through ranking, teaching, research, internationalisation and other academic indicators. However, an innovative operational approach has remained largely unaddressed in PHEIs. Therefore, PHEIs must adopt new business approaches and methods that have been developed by the business sectors, such as FI. Accordingly, this study seeks to fill the gap by examining the effect of IC on FI through ITC dimensions.

### Research gaps

Four research gaps ( Table 5) identified in this study were as follows:

**Gap 1.** PHEIs under resources constraint must emphasise utilising internal and external knowledge to build learning skills in a frugal environment, so that they may continue to deliver value-added service to their clients and increase shareholder wealth. Scholars have found that IC has a crucial role in generating innovation in a firm. ^39^^-^^42^ Thus, further investigations that evaluate the impact of IC are necessary to ensure sustained and improved firm performance using new context or perspectives, i.e., FI. Therefore, future research must address this significant issue by examining the extent to which IC in PHEIs can develop capabilities to fulfil the criteria identified in FI.

**Gap 2:** Technology and IT management will increase IC utilisation by exploiting technology in information management and processing, to enhance business capabilities and generate benefits for the organisation. ^43^^,^^44^ Hence, the effectiveness of IC management and utilisation is driven by the effectiveness of ITC and subsequently facilitate creation of value.

**Gap 3:** As IT becomes a fundamental business operation resource, employees with superior business knowledge would be able to utilise viable IT solutions and use their specialised technical skills to realign business strategies to meet the needs in a dynamic business environment, by creating business procedures and cost-feasible frameworks to achieve competitive advantage. ^45^^-^^47^ Many studies have revealed the relationship between ITC and competitive advantage. ^45^^,^^48^^,^^49^ Meanwhile, ^50^^,^^51^ argued that financial performance is higher for firms with higher digital innovation capabilities. Therefore, the ability of ITC to influence FI in PHEI must be emphasised.

**Figure 8.**
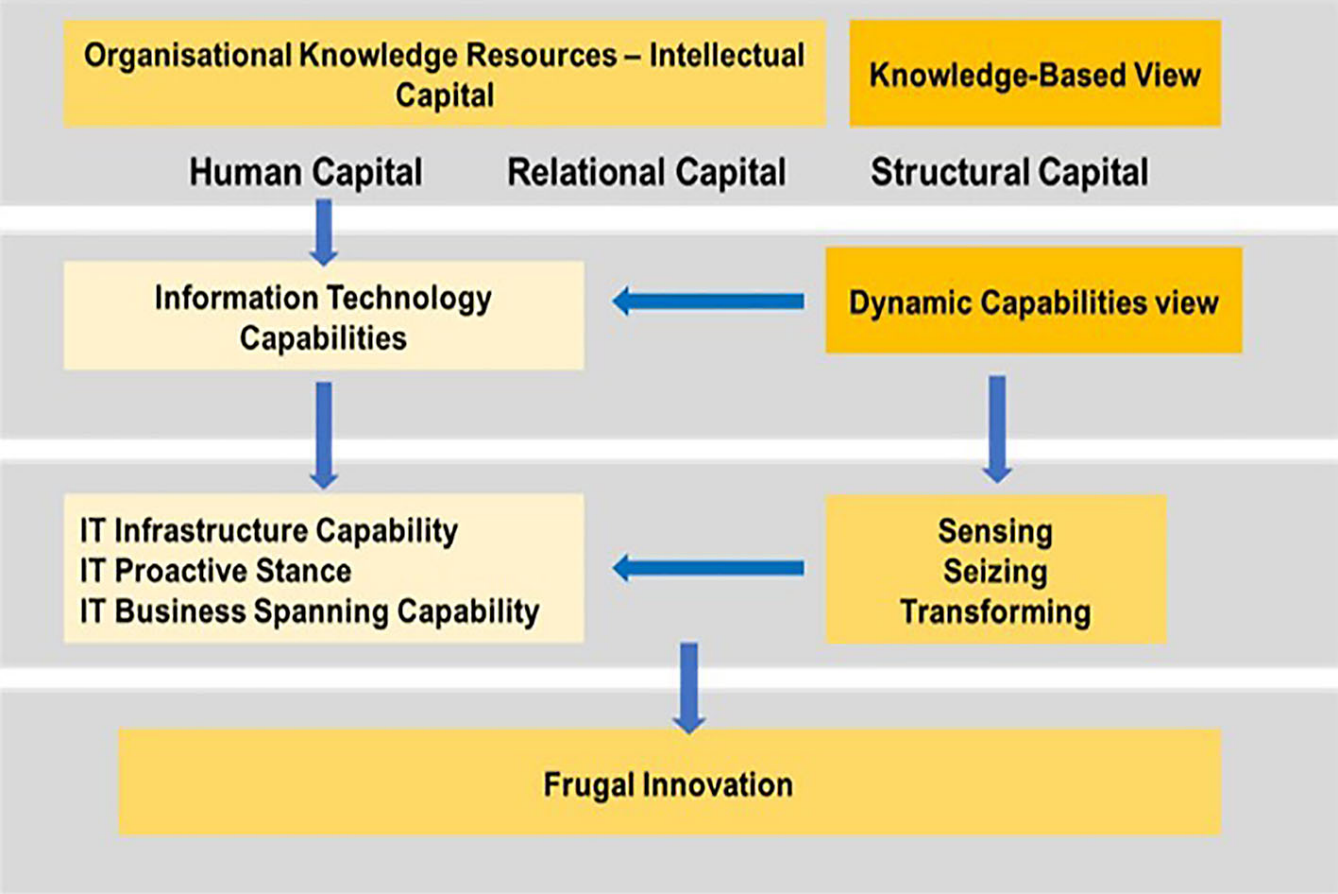
Conceptual framework.

**Figure 9.**
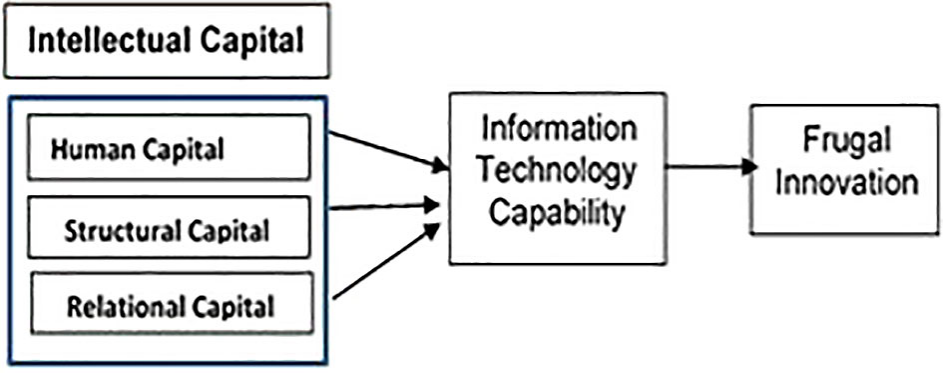
Theoretical framework.

**Gap 4:** As no studies focused on the effect of IC on FI through IT capability, the rationale behind the mediation effect of ITC is that a firm with a strong IC management is in a better position to deliver ITC to satisfy customers and optimise their resources to ‘produce more with less resources’ for cost efficiency. This situation mainly arises because of the high possibility that the relationship between IC and FI is not exclusively direct. Conceptually, ITC plays an important role in PHEI that has the attributes to gather all organisational knowledge resources and convert them to achieve FI (refer to Figure 8). Hence, a PHEI must have the capacity to effectively utilise its knowledge resources to generate dynamic capabilities that allow businesses to respond rapidly to changes in the environment. ^14^ The prevailing knowledge created from IC can create an opportunity to be ‘sensed and transformed’ to influence ITC, to enhance organisational efficiency and effectiveness through FI. As such, ITC can play a mediating role by translating IC into better performance to achieve FI ( Figures 8 and 9).

**Table 5.** Research gaps.

Intellectual Capital (IC) And Frugal Innovation (FI) In HEIs	Intellectual Capital (IC) And IT Capabilities (ITC)	IT Capabilities (ITC) And Frugal Innovation (FI)	Mediating Effect of It Capabilities Between Intellectual Capital (IC) And Frugal Innovation (FI)
Less Explored Context Inconclusive results between IC and organizational performance and innovation There is hardly any research that focuses on IC and Frugal innovation in the context of PHEI	Limited Research on IC and ITC within PHEI context There is a need to include some new indicators in dynamic business world such as information technology Capability (ITC) ^88^^,^^89^	Limited Findings ITC has not been used as one of the tools necessary in supporting Frugal innovation achievement in PHEI The investigation on Frugal innovation in higher education context still lacking ^12^^,^^90^^,^^91^	Less Explored Context The investigation of the mediating effect of ITC between Intellectual Capital and Frugal innovation in PHEI has not been explored

### Future research

The authors engaged in examining the relationship among IC, IT capability and FI, possibly may consider the main findings of this paper to examine the opportunities for future research. The two major themes for future articles include

• Theme 1: Evaluation model of IC, IT capability and FI in PHEIs
• Theme 2: Investigating the methodologies.
• Theme 3: Empirical analysis and developing measurement metrics for each variable.

This study is limited by the number of keywords selected. Keyword selection is based on research focus. However, it would be possible to obtain more articles if the keywords were expanded to fields of study that are not specific in nature, such as PHEI. This could possibly lead to a publication bias.

## Conclusions

As mentioned in the Introduction, research that explores the relationships between organisational IC dimensions, ITC and FI in the PHEIs in Malaysia remains insufficient. Thus, PHEIs should concentrate more on core activities, improve industry integration, engage with local and international communities, and increase operational efficiency. This study aims to inform IC scholars about the research gaps in the literature published from 1990 to 2020 regarding IC applications to achieve FI. The findings of this review suggest three major issues: firstly, an urgent need exists for scholars to streamline the use of concepts pertaining to FI in PHEIs. Secondly, more work is required to ascertain if IC and ITC share similar goals or otherwise to achieve FI. Finally, the use of the FI approach and its relationship with IC and ITC remains unexplored in the literature. Therefore, the extent to which the IC dimension interacts with FI in the PHEI context could be further explored. Finally, additional empirical research is needed to fully comprehend the relationship between IC, ITC and FI in the context of PHEIs.

## Data availability

### Underlying data

All data underlying the results is available as part of the article and no additional source data are required.

### Extended data

Figshare: Data file.xlsx, https://doi.org/10.6084/m9.figshare.14881437.v1. ^92^

This project contains the following underlying data:

- Data analysis: theories, type of papers, methods and findings

## Reporting guidelines

PRISMA checklist for “Unleashing frugal innovation in private higher education institutions via intellectual capital: a systematic literature review”, URL/DOI: https://doi.org/10.6084/m9.figshare.16726282. ^93^

Data are available under the terms of the Creative Commons Attribution 4.0 International license (CC-BY 4.0).
