# Supplementary material for: Unleashing frugal innovation in private higher education institutions via intellectual capital and Information technology capability: a systematic literature review
Source: F1000Res. 2022 May 20;10:1109. Originally published 2021 Nov 3. [Version 2] doi: 10.12688/f1000research.73329.2 (PMC9152465; doi:10.12688/f1000research.73329.2)
Supplement: Peer review history

# Peer review: Reviewer response for version 2

**DOI:** 10.5256/f1000research.134168.r138483

**Published:** 2022-05-30

**Authors:** Logaiswari Indiran

**Affiliations:** 1 Universiti Teknologi Malaysia, Johor Bahru, Malaysia

I would like to approve after going through the new version.

Are the rationale for, and objectives of, the Systematic Review clearly stated?

Yes

Is the statistical analysis and its interpretation appropriate?

Yes

Are sufficient details of the methods and analysis provided to allow replication by others?

Yes

Are the conclusions drawn adequately supported by the results presented in the review?

Yes

Reviewer Expertise:

NA

I confirm that I have read this submission and believe that I have an appropriate level of expertise to confirm that it is of an acceptable scientific standard.

# Peer review: Reviewer response for version 2

**DOI:** 10.5256/f1000research.134168.r138482

**Published:** 2022-05-27

**Authors:** Fivi Anggraini

**Affiliations:** 1 Faculty of Economics and Business, Universitas Bung Hatta, Padang, Indonesia

I have gone through this manuscript. I believe that the authors have revised the manuscript accordingly. Therefore, it resulted in an improved manuscript and is suitable for publication in this journal.

Are the rationale for, and objectives of, the Systematic Review clearly stated?

No

Is the statistical analysis and its interpretation appropriate?

Not applicable

Are sufficient details of the methods and analysis provided to allow replication by others?

Yes

Are the conclusions drawn adequately supported by the results presented in the review?

No

Reviewer Expertise:

Management accounting, intellectual capital

I confirm that I have read this submission and believe that I have an appropriate level of expertise to confirm that it is of an acceptable scientific standard.

# Peer review: Reviewer response for version 1

**DOI:** 10.5256/f1000research.76973.r99071

**Published:** 2022-02-18

**Authors:** Logaiswari Indiran

**Affiliations:** 1 Universiti Teknologi Malaysia, Johor Bahru, Malaysia

**General Comments:**

 Despite the fact that this is an intriguing study, the authors have obtained a unique dataset through the a comprehensive literature. On the whole, the paper is well-written and well-structured. However, I believe the study has several flaws, particularly on the reason for this research to be conducted, mainly on the importance/need of the relationship between IC, ITC and FI, and how the findings contribute significance contribution to the theoretical gaps.

**Introduction:**

 The problem statement is too general without any supporting detail. The reason for introducing IC and ITC towards FI is not clear.

**Method:**

 I would advise removing Figure 4, 5, and 6 as they are not suitable to systematic literature review study. Discussion for Figure 8 is not clear.

**Result and Discussion:**

 The analysis and discussion of the finding is too general and did not demonstrate any contribution to the theoretical gap. Need further discussion on the research gaps derived from Table 5.

**Summary:**

 I would advise adding details on the theoretical and contextual contribution of this study in the summary. Example: how is it significant to the existing knowledge and how it plays its role in context?

Are the rationale for, and objectives of, the Systematic Review clearly stated?

Yes

Is the statistical analysis and its interpretation appropriate?

Yes

Are sufficient details of the methods and analysis provided to allow replication by others?

Yes

Are the conclusions drawn adequately supported by the results presented in the review?

Yes

Reviewer Expertise:

Intellectual capital

I confirm that I have read this submission and believe that I have an appropriate level of expertise to confirm that it is of an acceptable scientific standard, however I have significant reservations, as outlined above.

# Response

**Published:** 2022-05-17

**Authors:** Jayamalathi Jayabalan

**Affiliations:** University Tunku Abdul Rahman, Malaysia

1. Problem statement: included more clear and with contextual detail to establish why it is important to include ITC

 2. Findings: Figure 4,5,6, and 7 is only added for descriptive analysis purposes as it might be useful for future research

 3. Results and discussion: Provided more explanations on the  major findings. Commented more on the significance of the studies and overall interpretation of the results 

 4. Summary: Provided detailed discussion to strengthen the section

# Peer review: Reviewer response for version 1

**DOI:** 10.5256/f1000research.76973.r119603

**Published:** 2022-02-03

**Authors:** Fivi Anggraini

**Affiliations:** 1 Faculty of Economics and Business, Universitas Bung Hatta, Padang, Indonesia

1. **General:**
  In general, this research is very interesting. The author has reviewed the literature extensively to produce this paper. However, there are some things that need to be improved so that this paper is more valuable for indexing.
2. **Title:**
  The title is incomplete because the author did not include information technology capabilities (ITC) variables.
3. **Introduction:**
  The research problem is not sharp to portray the issues. In fact, the argument is also not clear on why the author uses three variables in this study.
4. **Method:**
  What is the reason for the authors making statistics in Figures 4, 5, 6, and 7? In my opinion, the data is not related to the research objectives, because this research is a just systematic literature review, not investigational research.
5. **Results and discussion:**
  The discussion is very shallow; the authors have reviewed a lot of literature, but the discussion of the results is less in-depth.
  The intended research gap (in table 5) is not clear. Need to write down the author's analysis.
6. **Summary:**
  I think the authors failed to conclude significant findings as to the new theory in this study

What is the reason the authors concluded the frugal innovation, intellectual capital, and ITC insufficient? Need justification on the matters.

Are the rationale for, and objectives of, the Systematic Review clearly stated?

No

Is the statistical analysis and its interpretation appropriate?

Not applicable

Are sufficient details of the methods and analysis provided to allow replication by others?

Yes

Are the conclusions drawn adequately supported by the results presented in the review?

No

Reviewer Expertise:

management accounting, intellectual capital

I confirm that I have read this submission and believe that I have an appropriate level of expertise to confirm that it is of an acceptable scientific standard, however I have significant reservations, as outlined above.

# Response

**Published:** 2022-05-17

**Authors:** Jayamalathi Jayabalan

**Affiliations:** University Tunku Abdul Rahman, Malaysia

1. Title: Title is changed according to the suggestion given to include information technology Capabilities
2. Problem statement: included more clear and with contextual detail to establish why it is important
3. Findings: Figure 4,5,6, and 7 is only added for descriptive analysis purposes as it might be useful for future research
4. Results and discussion: Provided more explanations on the  major findings 
5. Summary: Provided detailed discussion to strengthen the sections
